# Dataset on the energy performance of atrium type hotel buildings

**DOI:** 10.1016/j.dib.2018.01.040

**Published:** 2018-01-31

**Authors:** Milica Vujosevic, Aleksandra Krstic-Furundzic

**Affiliations:** a Institute of Architecture and Urban & Spatial Planning of Serbia, Bulevar kralja Aleksandra 73/II, 11000 Belgrade, Serbia; b Faculty of Architecture, University of Belgrade, Bulevar kralja Aleksandra 73/II, 11000 Belgrade, Serbia

**Keywords:** Hotel, Atrium, Energy performance, Numerical simulation, Heating and cooling demands

## Abstract

The data presented in this article are related to the research article entitled “The Influence of Atrium on Energy Performance of Hotel Building” (Vujosevic and Krstic-Furundzic, 2017) [1], which describes the annual energy performance of atrium type hotel building in Belgrade climate conditions, with the objective to present the impact of the atrium on the hotel building's energy demands for space heating and cooling. This dataset is made publicly available to show energy performance of selected hotel design alternatives, in order to enable extended analyzes of these data for other researchers.

**Specifications Table**

**Table:** 

Subject area	*Architecture, Energy Efficiency*
More specific subject area	*Heating and cooling demands of atrium type hotel buildings*
Type of data	*Tables, figures, text file*
How data was acquired	*Building energy modeling in OpenStudio software*
	*Computer simulation using EnergyPlus software*
Data format	*Raw*
Experimental factors	*The four hypothetical model alternatives of atrium type hotel building, and four orientations of one model alternative, were examined and compared in order to find the most energy efficient solution*
Experimental features	*The optimal hotel design alternative were determined*
Data source location	*Belgrade, Serbia, 44.7866*°*N, 20.4489*°*E*
Data accessibility	*Data are available with this article*
Related research article	*M. Vujosevic, A. Krstic-Furundzic, The influence of atrium on energy performance of hotel building, Energy Build. (156), 2017, 140–150 (Elsevier)*

**Value of the data**

• The detailed thermal loading data provided for design alternatives of atrium type hotel buildings in Belgrade would give designers a better understanding of hotel energy demands.
• The building energy modeling and simulation is performed using EnergyPlus simulation software, and could be compared to other studies that used different simulation techniques.
• The data presented in this article could be used for analyzing the facilities located in the climatic conditions similar to Belgrade climate.
• Atrium design alternatives could be used as a basis for further research and development of hotel building envelopes.

## 1 Data

The data in this article present input data and schedules for the building energy simulation of design alternatives of atrium type hotel building (presented in Table 1), and detailed overview of all simulation results for each alternative and each thermal zone (presented in Table 2, Table 3, Table 4, Table 5, Table 6, Table 7, Table 8).

**Table 1:** Large hotel hourly operation schedules (work day – WD, Saturday – Sat, Sunday – Sun).

**Schedule**		Day	1	2	3	4	5	6	7	8	9	10	11	12	13	14	15	16	17	18	19	20	21	22	23	24
Always on		All	1	1	1	1	1	1	1	1	1	1	1	1	1	1	1	1	1	1	1	1	1	1	1	1
																										
Hotel Building	Equipment	WD	0.3	0.25	0.2	0.2	0.2	0.3	0.5	0.6	0.2	0.	0.35	0.35	0.35	0.35	0.35	0.35	0.35	0.35	0.7	0.9	0.95	0.9	0.7	0.4
		Sat	0.3	0.3	0.2	0.2	0.2	0.2	0.4	0.4	0.5	0.5	0.4	0.35	0.35	0.35	0.35	0.35	0.35	0.35	0.7	0.8	0.8	0.8	0.7	0.4
		Sun	0.4	0.4	0.3	0.3	0.3	0.3	0.4	0.5	0.5	0.4	0.4	0.4	0.4	0.3	0.3	0.3	0.3	0.3	0.6	0.8	0.9	0.7	0.6	0.4
																								
	Lighting	WD	0.2	0.15	0.1	0.1	0.1	0.2	0.4	0.5	0.4	0.4	0.25	0.25	0.25	0.25	0.25	0.25	0.25	0.25	0.6	0.8	0.9	0.8	0.6	0.3
		Sat	0.2	0.1	0.1	0.1	0.1	0.3	0.3	0.4	0.4	0.3	0.25	0.25	0.25	0.25	0.25	0.25	0.25	0.6	0.7	0.7	0.7	0.6	0.3	0.2
		Sun	0.3	0.2	0.2	0.2	0.2	0.3	0.4	0.4	0.3	0.3	0.3	0.3	0.2	0.2	0.2	0.2	0.2	0.5	0.7	0.8	0.6	0.5	0.3	0.3
																								
	Occupancy	WD	0.9	0.9	0.9	0.9	0.9	0.9	0.7	0.4	0.4	0.2	0.2	0.2	0.2	0.2	0.2	0.3	0.5	0.5	0.5	0.7	0.7	0.8	0.9	0.9
		Sat	0.9	0.9	0.9	0.9	0.9	0.9	0.7	0.5	0.5	0.3	0.3	0.3	0.3	0.3	0.3	0.3	0.3	0.5	0.6	0.6	0.6	0.7	0.7	0.7
		Sun	0.7	0.7	0.7	0.7	0.7	0.7	0.7	0.7	0.5	0.5	0.5	0.3	0.3	0.2	0.2	0.2	0.3	0.4	0.4	0.6	0.6	0.8	0.8	0.8
																									
Guest Room	Equipment	WD	0.2	0.2	0.2	0.2	0.2	0.2	0.62	0.9	0.43	0.43	0.26	0.26	0.26	0.26	0.26	0.26	0.26	0.51	0.51	0.49	0.66	0.7	0.35	0.2
		Sat. Sun	0.2	0.2	0.2	0.2	0.2	0.2	0.3	0.62	0.9	0.62	0.29	0.29	0.29	0.29	0.29	0.29	0.29	0.43	0.51	0.49	0.66	0.7	0.35	0.2
																								
	Lighting	WD	0.22	0.17	0.11	0.11	0.11	0.22	0.44	0.56	0.44	0.44	0.28	0.28	0.28	0.28	0.28	0.28	0.28	0.28	0.67	0.89	1	0.89	0.67	0.33
		Sat. Sun	0.26	0.26	0.11	0.11	0.11	0.11	0.41	0.41	0.56	0.56	0.41	0.33	0.33	0.33	0.33	0.33	0.33	0.33	0.85	1	1	1	0.85	0.41
																								
	Occupancy	WD	0.65	0.65	0.65	0.65	0.65	0.65	0.5	0.28	0.28	0.13	0.13	0.13	0.13	0.13	0.13	0.2	0.35	0.35	0.35	0.5	0.5	0.58	0.65	0.65
		Sat. Sun	0.65	0.65	0.65	0.65	0.65	0.65	0.5	0.34	0.34	0.2	0.2	0.2	0.2	0.2	0.2	0.2	0.2	0.34	0.35	0.65	0.65	0.5	0.5	0.5
																									
Loby	Occupancy	WD	0.1	0.1	0.1	0.1	0.1	0.3	0.7	0.7	0.7	0.7	0.2	0.2	0.2	0.2	0.2	0.2	0.4	0.4	0.2	0.2	0.2	0.2	0.1	0.1
		Sat. Sun	0.1	0.1	0.1	0.1	0.1	0.1	0.3	0.7	0.7	0.7	0.2	0.2	0.2	0.2	0.2	0.2	0.2	0.2	0.2	0.2	0.2	0.2	0.1	0.1
																								
Commercial area	Equipment	WD	0.4	0.4	0.4	0.4	0.4	0.4	0.4	0.4	0.9	0.9	0.9	0.9	0.8	0.9	0.9	0.9	0.9	0.8	0.6	0.6	0.5	0.5	0.4	0.4
		Sat	0.3	0.3	0.3	0.3	0.3	0.3	0.4	0.4	0.5	0.5	0.5	0.5	0.5	0.5	0.35	0.35	0.35	0.3	0.3	0.3	0.3	0.3	0.3	0.3
		Sun	0.3	0.3	0.3	0.3	0.3	0.3	0.3	0.3	0.3	0.3	0.3	0.3	0.3	0.3	0.3	0.3	0.3	0.3	0.3	0.3	0.3	0.3	0.3	0.3
																								
	Lighting	WD	0.05	0.05	0.05	0.05	0.05	0.1	0.1	0.3	0.9	0.9	0.9	0.9	0.9	0.9	0.9	0.9	0.9	0.7	0.5	0.5	0.3	0.3	0.1	0.05
		Sat	0.05	0.05	0.05	0.05	0.05	0.05	0.1	0.1	0.5	0.5	0.5	0.5	0.5	0.5	0.15	0.15	0.15	0.05	0.05	0.05	0.05	0.05	0.05	0.05
		Sun	0.05	0.05	0.05	0.05	0.05	0.05	0.05	0.05	0.05	0.05	0.05	0.05	0.05	0.05	0.05	0.05	0.05	0.05	0.05	0.05	0.05	0.05	0.05	0.05
																									
	Occupancy	WD	0	0	0	0	0	0	0.1	0.2	0.95	0.95	0.95	0.95	0.5	0.95	0.95	0.95	0.95	0.7	0.4	0.4	0.1	0.1	0.05	0.05
		Sat	0	0	0	0	0	0	0.1	0.1	0.5	0.5	0.5	0.5	0.5	0.5	0.1	0.1	0.1	0	0	0	0	0	0	0
		Sun	0	0	0	0	0	0	0	0	0	0	0	0	0	0	0	0	0	0	0	0	0	0	0	0

**Table 2:** Detailed overview of energy performance parameters – Alternative 1.

Alternative 1: the building with an open inner courtyard - without the atrium													
Thermal zone	Area [m^2^]	Volume [m^3^]	HVAC Input Sensible Air Heating [GJ]	HVAC Input Sensible Air Cooling [GJ]	People Sensible Heat Addition [GJ]	Lights Sensible Heat Addition [GJ]	Equipment Sensible Heat Addition [GJ]	Window Heat Addition [GJ]	Infiltration Heat Addition [GJ]	Opaque Surface Conduction and Other Heat Addition [GJ]	Window Heat Removal [GJ]	Infiltration Heat Removal [GJ]	Opaque Surface Conduction and Other Heat Removal [GJ]
Atrium	–	–	–	–	–	–	–	–	–	–	–	–	–
Corridor story 1	115.52	589.15	17.715	0	0.940	6.162	0	6.249	0.386	2.907	−6.788	−27.572	−0.001
Corridor story 2	274.72	1401.07	48.499	−1.691	2.127	14.655	0	61.375	0.161	0.002	−49.356	−71.701	−4.069
Corridor story 3	305.12	1141.15	31.506	−2.171	2.302	16.277	0	48.642	0.119	0.057	−35.5	−61.232	0
Corridor story 4	305.12	1141.15	30.516	−3.277	2.27	16.277	0	55.124	0.113	0.005	−36.044	−62.715	−2.264
Corridor story 5	305.12	1141.15	29.371	−4.544	2.242	16.277	0	61.763	0.107	0.002	−36.586	−64.096	−4.534
Corridor story 6	305.12	1141.15	27.847	−6.258	2.211	16.277	0	70.916	0.103	0.001	−37.031	−65.589	−8.476
Corridor story 7	305.12	1141.15	32.033	−8.722	2.199	16.277	0	83.551	0.102	0.001	−36.769	−66.032	−22.638
Commercial story 1	635.36	3240.34	49.815	−111.950	147.180	93.052	33.897	145.174	2.741	0.005	−78.031	−200.900	−80.978
Office story 2	635.36	3240.34	88.175	−89.275	24.055	71.465	62.973	116.572	2.705	0.005	−56.561	−191.832	−28.277
Room story 3	604.96	2262.55	61.288	−73.252	21.466	70.997	51.636	70.468	1.873	0	−33.952	−142.096	−28.428
Room story 4	604.96	2262.55	59.546	−74.558	21.431	70.997	51.636	70.955	1.854	0	−34.108	−142.706	−25.047
Room story 5	604.96	2262.55	59.190	−75.889	21.408	70.997	51.636	71.250	1.836	0	−34.219	−143.206	−23.002
Room story 6	604.96	2262.55	59.429	−77.111	21.398	70.997	51.636	71.474	1.818	0	−34.289	−143.57	−21.782
Room story 7	604.96	2262.55	75.395	−76.623	21.523	70.997	51.636	71.954	1.804	0	−33.749	−142.601	−40.338
**Total Facility**	**6211.36**	**25489.39**	**670.325**	**−605.320**	**292.753**	**621.704**	**355.050**	**1005.466**	**15.723**	**2.985**	**−542.983**	**−1525.848**	**−289.834**

**Table 3:** Detailed overview of energy performance parameters – Alternative 2.

Alternative 2: the building with glazed atrium – the basic atrium type building – southwest orientation 33													
Thermal zone	Area [m^2^]	Volume [m^3^]	HVAC Input Sensible Air Heating [GJ]	HVAC Input Sensible Air Cooling [GJ]	People Sensible Heat Addition [GJ]	Lights Sensible Heat Addition [GJ]	Equipment Sensible Heat Addition [GJ]	Window Heat Addition [GJ]	Infiltration Heat Addition [GJ]	Opaque Surface Conduction and Other Heat Addition [GJ]	Window Heat Removal [GJ]	Infiltration Heat Removal [GJ]	Opaque Surface Conduction and Other Heat Removal [GJ]
Atrium	693.12	16,242.21	602.187	−350.912	157.727	81.378	40.044	1029.683	13.364	0.003	−269.961	−916.353	−387.158
Corridor story 1	115.52	589.15	9.790	−0.008	0.907	6.165	0	0	0.204	12.552	0	−29.610	0
Corridor story 2	274.72	1401.07	11.398	−5.511	2.003	14.661	0	0	0.130	58.074	0	−80.756	0
Corridor story 3	305.12	1141.15	7.542	−4.603	2.201	16.284	0	6.131	0.104	42.090	−2.699	−67.048	0
Corridor story 4	305.12	1141.15	7.566	−5.037	2.195	16.284	0	6.164	0.102	42.850	−2.706	−67.417	0
Corridor story 5	305.12	1141.15	7.594	−5.439	2.190	16.284	0	6.182	0.101	43.575	−2.713	−67.771	0
Corridor story 6	305.12	1141.15	7.687	−5.765	2.186	16.284	0	6.193	0.099	44.060	−2.717	−68.027	0
Corridor story 7	305.12	1141.15	10.558	−5.269	2.204	16.284	0	6.233	0.098	39.720	−2.673	−67.155	0
Commercial story 1	635.36	3240.34	37.238	−113.076	145.808	93.092	33.897	115.598	2.741	0.003	−59.318	−203.750	−52.228
Office story 2	635.36	3240.34	82.538	−92.006	23.939	71.465	62.973	116.067	2.705	0.005	−57.234	−193.410	−17.037
Room story 3	604.96	2262.55	56.340	−74.786	21.368	71.028	51.636	70.257	1.870	0	−34.277	−143.061	−20.376
Room story 4	604.96	2262.55	54.962	−74.806	21.351	71.028	51.636	70.789	1.852	0	−34.380	−143.517	−18.913
Room story 5	604.96	2262.55	55.016	−75.036	21.349	71.028	51.636	71.130	1.834	0	−34.427	−143.817	−18.713
Room story 6	604.96	2262.55	55.832	−74.971	21.365	71.028	51.636	71.413	1.817	0	−34.418	−143.940	−19.762
Room story 7	604.96	2262.55	72.243	−73.285	21.518	71.028	51.636	71.953	1.803	0	−33.791	−142.708	−40.398
**Total Facility**	**6904.48**	**41,731.60**	**1078.492**	−**960.507**	**448.310**	**703.316**	**395.094**	**1647.792**	**28.824**	**282.932**	−**571.316**	−**2478.340**	−**574.585**

**Table 4:** Detailed overview of energy performance parameters – Alternative 3.

**Alternative 3: the building with glazed atrium and enhanced natural ventilation in atrium during summer**													
Thermal zone	Area [m^2^]	Volume [m^3^]	HVAC Input Sensible Air Heating [GJ]	HVAC Input Sensible Air Cooling [GJ]	People Sensible Heat Addition [GJ]	Lights Sensible Heat Addition [GJ]	Equipment Sensible Heat Addition [GJ]	Window Heat Addition [GJ]	Infiltration Heat Addition [GJ]	Opaque Surface Conduction and Other Heat Addition [GJ]	Window Heat Removal [GJ]	Infiltration Heat Removal [GJ]	Opaque Surface Conduction and Other Heat Removal [GJ]
Atrium	693.12	16,242.21	630.526	−296.785	162.664	81.378	40.044	1035.780	33.016	0.003	−262.611	−1074.802	−349.211
Corridor story 1	115.52	589.15	9.790	−0.004	0.913	6.165	0	0	0.238	12.199	0	−29.300	0
Corridor story 2	274.72	1401.07	11.398	−4.134	2.041	14.661	0	0	0.130	54.694	0	−78.790	0
Corridor story 3	305.12	1141.15	7.542	−3.428	2.240	16.284	0	6.180	0.104	39.288	−2.653	−65.557	0
Corridor story 4	305.12	1141.15	7.566	−3.777	2.234	16.284	0	6.213	0.102	39.974	−2.660	−65.935	0
Corridor story 5	305.12	1141.15	7.594	−4.104	2.229	16.284	0	6.230	0.101	40.633	−2.667	−66.299	0
Corridor story 6	305.12	1141.15	7.687	−4.371	2.225	16.284	0	6.241	0.099	41.069	−2.671	−66.563	0
Corridor story 7	305.12	1141.15	10.558	−4.010	2.242	16.284	0	6.281	0.098	36.884	−2.627	−65.709	0
Commercial story 1	635.36	3240.34	37.238	−110.737	145.896	93.092	33.897	115.694	2.741	0.003	−59.223	−203.573	−55.024
Office story 2	635.36	3240.34	82.54	−90.120	23.947	71.465	62.973	116.164	2.705	0.005	−57.126	−193.141	−19.407
Room story 3	604.96	2262.55	56.34	−72.703	21.384	71.028	51.636	70.304	1.870	0	−34.229	−142.944	−22.686
Room story 4	604.96	2262.55	54.962	−72.742	21.366	71.028	51.636	70.834	1.852	0	−34.333	−143.405	−21.198
Room story 5	604.96	2262.55	55.016	−72.979	21.364	71.028	51.636	71.175	1.834	0	−34.381	−143.706	−20.988
Room story 6	604.96	2262.55	55.832	−72.924	21.381	71.028	51.636	71.458	1.817	0	−34.371	−143.827	−22.030
Room story 7	604.96	2262.55	72.243	−71.375	21.536	71.028	51.636	72.000	1.803	0	−33.743	−142.579	−42.548
**Total Facility**	**6904.48**	**41,731.60**	**1106.833**	−**884.193**	**453.662**	**703.316**	**395.094**	**1654.553**	**48.510**	**264.753**	−**563.296**	−**2626.129**	−**553.091**

**Table 5:** Detailed overview of energy performance parameters – Alternative 4.

**Alternative 4: the building with atrium equipped with shading devices**													
Thermal zone	Area [m^2^]	Volume [m^3^]	HVAC Input Sensible Air Heating [GJ]	HVAC Input Sensible Air Cooling [GJ]	People Sensible Heat Addition [GJ]	Lights Sensible Heat Addition [GJ]	Equipment Sensible Heat Addition [GJ]	Window Heat Addition [GJ]	Infiltration Heat Addition [GJ]	Opaque Surface Conduction and Other Heat Addition [GJ]	Window Heat Removal [GJ]	Infiltration Heat Removal [GJ]	Opaque Surface Conduction and Other Heat Removal [GJ]
Atrium	693.12	16,242.21	702.054	−149.618	161.620	81.378	40.044	450.428	13.398	0.008	−243.626	−874.651	−181.028
Corridor story 1	115.52	589.15	11.302	0	0.936	6.165	0	0	0.414	9.144	0	−27.961	0
Corridor story 2	274.72	1401.07	15.166	−0.255	2.139	14.661	0	0	0.208	39.965	0	−71.885	0
Corridor story 3	305.12	1141.15	9.898	−0.248	2.347	16.284	0	6.089	0.154	28.071	−2.488	−60.106	0
Corridor story 4	305.12	1141.15	9.922	−0.295	2.342	16.284	0	6.123	0.145	28.405	−2.494	−60.433	0
Corridor story 5	305.12	1141.15	9.958	−0.335	2.338	16.284	0	6.147	0.138	28.701	−2.499	−60.731	0
Corridor story 6	305.12	1141.15	10.108	−0.371	2.335	16.284	0	6.155	0.132	28.801	−2.503	−60.941	0
Corridor story 7	305.12	1141.15	13.774	−0.406	2.346	16.284	0	6.209	0.133	24.534	−2.469	−60.405	0
Commercial story 1	635.36	3240.34	38.842	−104.283	146.746	93.092	33.897	116.214	2.741	0.002	−58.499	−201.770	−66.980
Office story 2	635.36	3240.34	85.065	−86.744	24.039	71.465	62.973	116.435	2.705	0.004	−56.595	−191.774	−27.568
Room story 3	604.96	2262.55	58.777	−69.355	21.487	71.028	51.636	70.513	1.872	0	−33.926	−141.970	−30.062
Room story 4	604.96	2262.55	57.394	−69.379	21.472	71.028	51.636	71.048	1.855	0	−34.021	−142.397	−28.635
Room story 5	604.96	2262.55	57.485	−69.588	21.471	71.028	51.636	71.393	1.837	0	−34.064	−142.685	−28.513
Room story 6	604.96	2262.55	58.337	−69.533	21.486	71.028	51.636	71.675	1.819	0	−34.057	−142.818	−29.574
Room story 7	604.96	2262.55	74.966	−68.341	21.625	71.028	51.636	72.193	1.806	0	−33.474	−141.727	−49.712
**Total Facility**	**6904.48**	**41,731.60**	**1213.048**	−**688.750**	**454.729**	**703.316**	**395.094**	**1070.623**	**29.358**	**187.634**	−**540.713**	−**2382.254**	−**442.072**

**Table 6:** Detailed overview of energy performance parameters – Alternative 2 southeast orientation.

**Alternative 2: the building with glazed atrium – the basic atrium type building – southeast orientation 303**													
Thermal zone	Area [m^2^]	Volume [m^3^]	HVAC Input Sensible Air Heating [GJ]	HVAC Input Sensible Air Cooling [GJ]	People Sensible Heat Addition [GJ]	Lights Sensible Heat Addition [GJ]	Equipment Sensible Heat Addition [GJ]	Window Heat Addition [GJ]	Infiltration Heat Addition [GJ]	Opaque Surface Conduction and Other Heat Addition [GJ]	Window Heat Removal [GJ]	Infiltration Heat Removal [GJ]	Opaque Surface Conduction and Other Heat Removal [GJ]
Atrium	693.12	16,242.21	615.661	−365.852	157.544	81.378	40.044	1019.596	13.364	0.002	−264.898	−913.801	−383.038
Corridor story 1	115.52	589.15	10.047	−0.014	0.906	6.165	0	0	0.197	12.297	0	−29.598	0
Corridor story 2	274.72	1401.07	12.033	−6.180	1.996	14.661	0	0	0.133	57.831	0	−80.474	0
Corridor story 3	305.12	1141.15	7.974	−5.147	2.194	16.284	0	6.587	0.106	41.474	−2.645	−66.827	0
Corridor story 4	305.12	1141.15	7.984	−5.436	2.190	16.284	0	6.623	0.104	42.085	−2.653	−67.180	0
Corridor story 5	305.12	1141.15	7.964	−5.689	2.186	16.284	0	6.641	0.102	42.705	−2.661	−67.531	0
Corridor story 6	305.12	1141.15	7.993	−5.907	2.184	16.284	0	6.655	0.100	43.128	−2.666	−67.772	0
Corridor story 7	305.12	1141.15	10.939	−5.366	2.202	16.284	0	6.697	0.099	38.680	−2.623	−66.911	0
Commercial story 1	635.36	3240.34	38.199	−117.415	145.955	93.092	33.897	118.093	2.741	0.003	−59.251	−203.425	−51.886
Office story 2	635.36	3240.34	82.332	−96.315	23.934	71.465	62.973	122.729	2.705	0.005	−57.463	−193.764	−18.595
Room story 3	604.96	2262.55	54.862	−71.665	21.335	71.028	51.636	69.486	1.868	0	−34.782	−143.313	−20.455
Room story 4	604.96	2262.55	53.363	−71.270	21.317	71.028	51.636	70.003	1.850	0	−34.889	−143.783	−19.255
Room story 5	604.96	2262.55	53.364	−71.392	21.315	71.028	51.636	70.335	1.833	0	−34.937	−144.087	−19.093
Room story 6	604.96	2262.55	54.157	−71.257	21.332	71.028	51.636	70.601	1.815	0	−34.925	−144.199	−20.188
Room story 7	604.96	2262.55	70.482	−69.681	21.493	71.028	51.636	71.141	1.800	0	−34.266	−142.895	−40.736
**Total Facility**	**6904.48**	**41,731.60**	**1087.353**	−**968.586**	**448.084**	**703.316**	**395.094**	**1645.188**	**28.817**	**278.209**	−**568.659**	−**2475.560**	−**573.247**

**Table 7:** Detailed overview of energy performance parameters – Alternative 2 northwest orientation.

**Alternative 2: the building with glazed atrium – the basic atrium type building – northwest orientation 123**													
Thermal zone	Area [m^2^]	Volume [m^3^]	HVAC Input Sensible Air Heating [GJ]	HVAC Input Sensible Air Cooling [GJ]	People Sensible Heat Addition [GJ]	Lights Sensible Heat Addition [GJ]	Equipment Sensible Heat Addition [GJ]	Window Heat Addition [GJ]	Infiltration Heat Addition [GJ]	Opaque Surface Conduction and Other Heat Addition [GJ]	Window Heat Removal [GJ]	Infiltration Heat Removal [GJ]	Opaque Surface Conduction and Other Heat Removal [GJ]
Atrium	693.12	16,242.21	669.881	−299.010	159.194	81.378	40.044	830.627	13.366	0.004	−264.664	−899.723	−331.092
Corridor story 1	115.52	589.15	10.442	−0.007	0.911	6.165	0	0	0.231	11.538	0	−29.281	0
Corridor story 2	274.72	1401.07	13.930	−4.169	2.047	14.661	0	0	0.145	50.782	0	−77.395	0
Corridor story 3	305.12	1141.15	9.110	−3.423	2.246	16.284	0	6.679	0.114	36.031	−2.592	−64.450	0
Corridor story 4	305.12	1141.15	9.111	−3.631	2.242	16.284	0	6.715	0.111	36.580	−2.600	−64.811	0
Corridor story 5	305.12	1141.15	9.018	−3.809	2.238	16.284	0	6.732	0.106	37.234	−2.608	−65.193	0
Corridor story 6	305.12	1141.15	8.947	−3.971	2.235	16.284	0	6.744	0.103	37.746	−2.614	−65.473	0
Corridor story 7	305.12	1141.15	12.165	−3.688	2.250	16.284	0	6.782	0.103	33.422	−2.575	−64.743	0
Commercial story 1	635.36	3240.34	35.032	−127.837	144.857	93.092	33.897	145.872	2.741	0.003	−59.731	−205.769	−62.154
Office story 2	635.36	3240.34	76.473	−107.127	23.678	71.465	62.973	148.877	2.705	0.005	−57.705	−195.852	−25.487
Room story 3	604.96	2262.55	51.521	−76.445	21.263	71.028	51.636	82.405	1.868	0	−34.498	−144.210	−24.568
Room story 4	604.96	2262.55	50.304	−75.839	21.25	71.028	51.636	82.844	1.85	0	−34.586	−144.625	−23.859
Room story 5	604.96	2262.55	50.321	−75.926	21.248	71.028	51.636	83.107	1.832	0	−34.634	−144.923	−23.689
Room story 6	604.96	2262.55	51.106	−75.755	21.267	71.028	51.636	83.328	1.815	0	−34.620	−145.025	−24.779
Room story 7	604.96	2262.55	67.065	−73.887	21.439	71.028	51.636	83.864	1.798	0	−33.945	−143.585	−45.413
**Total Facility**	**6904.48**	**41,731.60**	**1124.426**	−**934.524**	**448.365**	**703.316**	**395.094**	**1574.574**	**28.889**	**243.344**	−**567.373**	−**2455.06**	−**561.042**

**Table 8:** Detailed overview of energy performance parameters – Alternative 2 northeast orientation.

**Alternative 2: the building with glazed atrium – the basic atrium type building – northeast orientation 213**													
Thermal zone	Area [m^2^]	Volume [m^3^]	HVAC Input Sensible Air Heating [GJ]	HVAC Input Sensible Air Cooling [GJ]	People Sensible Heat Addition [GJ]	Lights Sensible Heat Addition [GJ]	Equipment Sensible Heat Addition [GJ]	Window Heat Addition [GJ]	Infiltration Heat Addition [GJ]	Opaque Surface Conduction and Other Heat Addition [GJ]	Window Heat Removal [GJ]	Infiltration Heat Removal [GJ]	Opaque Surface Conduction and Other Heat Removal [GJ]
Atrium	693.12	16,242.21	675.363	−276.051	159.187	81.378	40.044	779.342	13.368	0.004	−262.693	−897.279	−312.660
Corridor story 1	115.52	589.15	10.474	−0.003	0.914	6.165	0	0	0.248	11.342	0	−29.139	0
Corridor story 2	274.72	1401.07	13.973	−2.825	2.054	14.661	0	0	0.148	48.792	0	−76.803	0
Corridor story 3	305.12	1141.15	9.140	−2.412	2.255	16.284	0	6.214	0.116	35.002	−2.597	−64.002	0
Corridor story 4	305.12	1141.15	9.153	−2.713	2.249	16.284	0	6.247	0.112	35.654	−2.604	−64.382	0
Corridor story 5	305.12	1141.15	9.103	−2.984	2.243	16.284	0	6.263	0.109	36.375	−2.612	−64.781	0
Corridor story 6	305.12	1141.15	9.100	−3.200	2.239	16.284	0	6.273	0.107	36.901	−2.617	−65.086	0
Corridor story 7	305.12	1141.15	12.387	−3.011	2.253	16.284	0	6.309	0.107	32.653	−2.579	−64.403	0
Commercial story 1	635.36	3240.34	34.380	−121.307	144.730	93.092	33.897	141.322	2.741	0.003	−60.331	−206.218	−62.306
Office story 2	635.36	3240.34	74.561	−104.252	23.670	71.465	62.973	150.157	2.705	0.005	−58.247	−196.372	−26.660
Room story 3	604.96	2262.55	51.632	−80.307	21.255	71.028	51.636	87.294	1.868	0	−34.484	−144.279	−25.643
Room story 4	604.96	2262.55	50.637	−80.041	21.245	71.028	51.636	87.716	1.850	0	−34.562	−144.660	−24.849
Room story 5	604.96	2262.55	50.710	−80.221	21.245	71.028	51.636	87.967	1.832	0	−34.607	−144.947	−24.644
Room story 6	604.96	2262.55	51.522	−80.104	21.263	71.028	51.636	88.183	1.815	0	−34.594	−145.050	−25.698
Room story 7	604.96	2262.55	67.464	−78.087	21.433	71.028	51.636	88.705	1.799	0	−33.930	−143.632	−46.416
**Total Facility**	**6904.48**	**41,731.60**	**1129.601**	−**917.516**	**448.234**	**703.316**	**395.094**	**1541.990**	**28.925**	**236.732**	−**566.456**	−**2451.030**	−**548.876**

## 2 Experimental design, materials and methods

The created hypothetical model is “U” shaped building, with rooms surrounding a central courtyard from three sides. Following alternatives are subject to the numerical simulations, which give an insight of their energy performances: the building with an open inner courtyard - without the atrium, the building with glazed atrium – the basic atrium type building, the building with glazed atrium and enhanced natural ventilation in atrium during summer, and the building with atrium equipped with shading devices. All alternatives were simulated with southwest oriented atrium. The basic atrium type building is also simulated in three other orientations (southeast, northwest and northeast atrium orientation).

The research method includes the creation of a hypothetical model for the analysis, design of alternatives, application of numerical simulations using computer software, and comparative analyses of the selected results. The building energy simulation is carried out by OpenStudio SketchUp PlugIn – an interface that integrates with EnergyPlus simulation engine. Certain parameters in the particular model are changed (for example building orientation, enhanced infiltration, shading system) so that the observation of how the changes influence energy performance of the building could be conducted.
