# Supplementary material for: Dataset on the energy performance of atrium type hotel buildings
Source: Data Brief. 2018 Jan 31;17:1336–47. doi: 10.1016/j.dib.2018.01.040 (PMC5988298; doi:10.1016/j.dib.2018.01.040)
Supplement: Supplementary file 1 — Supplementary material. [file mmc1.docx]

Ref. No.: DIB-D-17-00961

Title: THE INFLUENCE OF THE ATRIUM ON THE ENERGY PERFORMANCE OF THE HOTEL BUILDING

Journal: Data In Brief

January 5, 2018

**Conflict of Interest**

Dear Editor,

We declare no conflict of interest.

Sincerely,

Dr. Milica Vujošević, corresponding author

Prof. Dr. Aleksandra Krstić-Furundžić, author
